# Use of Noninvasive Ventilation with Volume-Assured Pressure Support to Avoid Tracheostomy in Severe Obstructive Sleep Apnea

**DOI:** 10.1155/2018/4701736

**Published:** 2018-10-09

**Authors:** Montserrat Diaz-Abad, Amal Isaiah, Valerie E Rogers, Kevin D. Pereira, Anayansi Lasso-Pirot

**Affiliations:** ^1^Department of Medicine, University of Maryland School of Medicine, Baltimore, Maryland, USA; ^2^Department of Otorhinolaryngology-Head and Neck Surgery, University of Maryland School of Medicine, Baltimore, Maryland, USA; ^3^Department of Family and Community Health, University of Maryland School of Nursing, Baltimore, Maryland, USA; ^4^Department of Pediatrics, University of Maryland School of Medicine, Baltimore, Maryland, USA

## Abstract

Obstructive sleep apnea (OSA) is a common disorder in children but can occasionally present with life-threatening hypoxemia. Obesity is a significant risk factor for poor outcomes of OSA treatment. Continuous positive airway pressure (CPAP) is indicated in children who are not candidates for or have an unsatisfactory response to adenotonsillectomy. Children acutely at risk for significant morbidity with other therapies are candidates for a tracheostomy. An eight-year-old patient with morbid obesity and severe OSA refractory to CPAP therapy was treated successfully with a novel noninvasive ventilation (NIV) mode with volume-assured pressure support (VAPS) and avoided tracheostomy.

## 1. Introduction

Obstructive sleep apnea (OSA) is a common disorder in children, with a prevalence of 1.2–5.7% [1], but can occasionally present with life-threatening hypoxemia. Obesity and severe OSA are significant risk factors for poor outcomes of OSA treatment. Although adenotonsillectomy is considered first-line therapy for OSA [1], the prevalence of residual OSA in obese children after adenotonsillectomy ranges from 33% to 76% [2], and a high apnea-hypopnea index (AHI, events/h) predicts residual disease after surgery in nonobese children [3]. Continuous positive airway pressure (CPAP) is indicated in children who are not candidates for or have an unsatisfactory response to adenotonsillectomy [1]. Children acutely at risk for significant morbidity with other therapies are candidates for a tracheostomy [4]. We present a pediatric patient with morbid obesity and severe OSA refractory to CPAP therapy. A novel noninvasive ventilation (NIV) mode with volume-assured pressure support (VAPS) was used successfully to avoid tracheostomy.

## 2. Case Presentation

An eight-year-old male presented with loud snoring, witnessed apneas, restless sleep, mouth breathing, nocturnal enuresis, excessive daytime sleepiness, behavioral problems, and academic difficulties due to frequently falling asleep at school. Past medical history included 36-week gestation, hypertension, high-functioning autism spectrum disorder, and attention-deficit/hyperactivity disorder. Physical exam was significant for a body mass index (BMI) of 51.8 kg/m^2^ (BMI z-score 2.9), a blood pressure value of 105/80 mm Hg (systolic 53.1^th^ percentile-for-age/height, diastolic 93.7^th^ percentile), obese neck, 3+ tonsils, oropharyngeal crowding, hyperactivity, and blunted effect.

**Figure 1:**
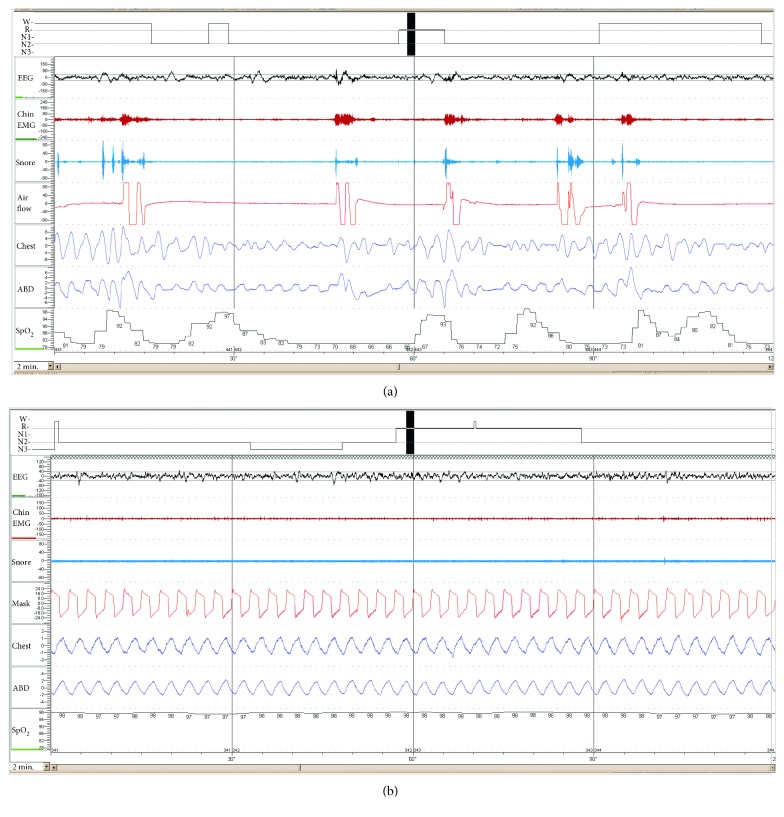
Baseline polysomnogram demonstrated severe obstructive sleep apnea and hypoxemia (a). There was a significant reduction of obstructive events and normalization of oxygenation during AVAPS-AE titration (b). Both figures shown in 2-minute intervals are of rapid eye movement sleep.

In-laboratory polysomnography revealed severe OSA, with AHI 138.2, sleep hypoventilation, and hypoxemia (Table 1 and Figure 1(a)). Given the severity of his sleep-disordered breathing and morbid obesity, the multidisciplinary pediatric sleep team concluded that the patient was a high-risk candidate for surgery with a very low probability of significant reduction in AHI after adenotonsillectomy. A trial of CPAP was recommended along with weight loss. Both tracheostomy and adenotonsillectomy were planned if these measures failed. During his titration polysomnogram, CPAP via nasal mask was titrated up to 19 cm H_2_O with only partial reduction of the AHI. Further increases in CPAP pressure were not tolerated.

Despite the pressure intolerance, the patient tolerated the mask well and felt better the following day. Alternative NIV treatment options were considered prior to proceeding with surgery, including VAPS mode with autotitrating expiratory positive airway pressure (EPAP), which has been used successfully in adults with coexisting OSA and hypoventilation syndromes [5]. NIV mode average VAPS with autotitrating EPAP (AVAPS-AE) with nasal mask was initiated in the clinic and continued for a one-week trial at home. The patient tolerated this well and reported feeling better rested during the home trial. A second titration study was performed one week later to optimize NIV settings. Immediate, substantial reduction of the AHI was achieved, with significant improvement in oxygenation, ventilation, and sleep quality (Table 1 and Figure 1(b)). A Trilogy® ventilator (Philips Respironics, Murrysville, PA, USA) was prescribed with AVAPS-AE mode and a nasal mask with the following settings: EPAP 5–16 cm H_2_O, pressure support 4–17 cm H_2_O, maximum pressure 25 cm H_2_O, inspiratory time 1.5 sec, rise time 3, tidal volume 390 ml (8 ml/kg), and breath rate 20/min.

In follow-up, data download showed good compliance with therapy and control of OSA, with 90% of days used, and average nightly use of 6.4 h without apneas. The father reported that the child was more active and was no longer falling asleep at school. In the clinic, he demonstrated normal mood and was fully awake, calmer, and more interactive. Further interventions focused on weight management, including an admission to an inpatient multidisciplinary weight reduction program, with plans for adenotonsillectomy if significant weight loss was achieved.

## 3. Discussion

This report demonstrates the effective use of NIV VAPS to treat OSA refractory to CPAP in a morbidly obese pediatric patient. This is a novel strategy for a child, resulting in a reduction of AHI from very severe (138.2) to moderate levels (9.7) and avoidance of tracheostomy. The child demonstrated good compliance and clinical response.

Although adenotonsillectomy is considered first-line therapy for childhood OSA [1], CPAP and bilevel PAP can be started in selected children with OSA who have not previously undergone adenotonsillectomy [2]. The marked severity of sleep-disordered breathing and coexisting morbid obesity in this patient increased his surgical risk [6], while decreasing the likelihood of benefit. Although studies have shown improvement in AHI in obese children after adenotonsillectomy [2, 7, 8], the prevalence of residual OSA ranged from 33% to 76%, [2] and subsequent weight gain was common [9]. In children with a BMI z-score >2.5, a mean reduction in AHI of only 10 events/h can be expected with adenotonsillectomy alone, while children whose BMI z-score exceeded 3 derived little improvement in AHI [8], suggesting that morbidly obese patients require a more complex approach to treatment than those of normal weight [8]. As tracheostomy was considered the next best option to manage this child's disease, a trial of NIV was attempted with the hope of avoiding this procedure which is also associated with morbidity and reduces quality of life significantly.

Compared to CPAP, AVAPS mode allows the clinician to set variable pressure support that self-adjusts to maintain target tidal volume despite varying respiratory mechanics, ventilatory control, upper airway patency, and respiratory muscle recruitment [10]. It also maintains stable ventilation that can adapt to disease progression in conditions such as neuromuscular disease [11]. Advanced devices add autotitrating EPAP and an automatic back-up rate based on the patient's spontaneous respiratory rate [10]. Autotitrating EPAP has similar effectiveness to fixed EPAP from an in-laboratory CPAP titration for treating coexisting OSA in adults with hypoventilation syndromes receiving NIV [5].

The use of NIV VAPS is uncommon in children, limited to two case reports in congenital central hypoventilation [12, 13] and one case report in myopathy [14]. This report suggests a novel use of this NIV mode. Our experience suggests that a trial of NIV VAPS with either a fixed or autotitrating EPAP may be considered as a potential treatment option in obese children with severe OSA who fail CPAP therapy prior to considering tracheostomy, where appropriate institutional and family support are available.

## Figures and Tables

**Table 1:** Polysomnography parameters at baseline and on CPAP and AVAPS-AE.

Parameter	Baseline	CPAP titration	AVAPS-AE titration
Total sleep time (min)	323.5	358.0	429.0
Sleep efficiency (%)	78.5	82.3	97.1
Sleep latency (min)	0.5	3.0	3.0
R latency (min)	175.5	238.8	132.5
Wake (min) (%)	88.5 (21.5)	77.0 (17.7)	13.0 (2.9)
N1 (min) (%)	1.0 (0.2)	0.0 (0.0)	0.0 (0.0)
N2 (min) (%)	238.5 (57.9)	175.5 (40.3)	268.0 (60.6)
N3 (min) (%)	63.5 (15.4)	127.0 (29.2)	72.5 (16.4)
R (min) (%)	20.5 (5.0)	55.5 (12.8)	88.5 (20.0)
Arousals index (arousals/h)	72.3	39.7	12.3
Periodic limb movement index (events/h)	0.0	0.0	0.0
Apnea-hypopnea index (events/h)	138.2	57.5	9.7
Obstructive apnea index (events/h)	122.0	44.1	0.1
Mean SpO_2_ (%)	92	94	97
Minimum SpO_2_ (%)	59	72	93
Time SpO_2_ ≤ 90% (min)	111.7	55.9	0.0
Baseline ETCO_2_ (mm Hg)	50	36	47
Maximum ETCO_2_ (mm Hg)	60	48	51
Time ETCO_2_ ≥ 50 (mm Hg) (min)	123.3	0.0	0.7

*Note*. On diagnostic (baseline) polysomnography, oxygen at 0.25 L/min via nasal cannula was added 40 min after sleep onset due to severe hypoxemia without rebound and maintained for the remainder of the study. CPAP and AVAPS-AE studies were done in room air. AVAPS-AE, average volume-assured pressure support with autotitrating expiratory positive airway pressure; CPAP, continuous positive airway pressure; R, rapid eye movement sleep; N, nonrapid eye movement sleep; SpO_2_, oxygen saturation by pulse oximetry; ETCO_2_, end-tidal carbon dioxide.
